# House sparrows’ (*Passer domesticus*) behaviour in a novel environment is modulated by social context and familiarity in a sex-specific manner

**DOI:** 10.1186/s12983-018-0267-8

**Published:** 2018-04-20

**Authors:** Beniamino Tuliozi, Gerardo Fracasso, Herbert Hoi, Matteo Griggio

**Affiliations:** 1 Department of Biology, University of Padova, Via U. Bassi 58/B, I-35131 Padova, Italy; 2 Evolutionary Ecology Group, Department of Biology, University of Antwerp, Universiteitsplein 1, B-2610 Wilrijk, Belgium; 3 Konrad Lorenz Institute of Ethology, Department of Integrative Biology and Evolution, University of Veterinary Medicine Vienna, Savoyenstrasse 1a, A-1160 Vienna, Austria

**Keywords:** Exploration, Familiarity, House sparrow, Invasive species, Novel environment, Open-field test, *Passer domesticus*, Personality, Sex-difference, Social behaviour

## Abstract

**Background:**

Exploratory behaviour is one of the best-investigated behavioural traits. However, little is known about how differences in familiarity, i.e. in the knowledge and previous experience with a companion can influence the exploration of a novel environment. However, to our knowledge, such a critical feature of the social environment has never been the target of a study relating it to exploratory behaviour in birds. Here we examined if familiarity with a conspecific could affect behavioural responses of individuals confronted with a novel environment. We recorded the latency to land on the ground, latency to feed, time spent feeding and number of sectors visited of 48 female and 48 male house sparrows (*Passer domesticus*) in an indoor aviary in three contexts: alone (individual context), with an unfamiliar and with a familiar same-sex companion.

**Results:**

House sparrows landed sooner on the ground when in the familiar context than when in the individual context. Birds in unfamiliar pairs followed each other less than familiar birds, but this difference diminished with time spent exploring. Moreover, males and females differed in their behavioural responses in the unfamiliar context. Females with a familiar companion landed sooner than when they were paired with an unfamiliar conspecific, whereas only the presence of a companion but not familiarity reduced males latency to land on the ground. Finally, when considering the unfamiliar context males had shorter latencies to forage and thus spent more time eating than females.

**Conclusions:**

The presence or absence of a companion and its familiarity with the focal individual influenced differently the behavioural responses of male and female house sparrows in a novel environment. As house sparrows are strongly sociable, the influence of the social environment is likely to be of paramount importance to understand the selective pressures acting on them, particularly in recently colonized areas with ephemeral food sources. Our results shed light on the complex influence that the social environment has on the behavioural responses of a cosmopolitan bird.

**Electronic supplementary material:**

The online version of this article (10.1186/s12983-018-0267-8) contains supplementary material, which is available to authorized users.

## Background

Behavioural responses to novel environments (such as exploratory behaviour and neophobia) are considered critical targets of selective pressures [1–3]. While animals exposed to unfamiliar environments generally perceive them as less predictable and more dangerous than familiar places and situations [4–6], they are often forced to explore, disperse and colonise new areas [7, 8]. However, exploratory traits are often investigated using animals in an individual context, while the presence of conspecifics can modulate the expression of behavioural responses, for example through social facilitation (change of rate of certain behavioural responses, sensu [9]).

Indeed, in recent years the social environment has been more and more often recognized to play an important role in shaping the evolution of various behavioural and physiological traits [10] and individuals facing a novel environment can gain various benefits from being in a group. The presence of conspecifics could result in social buffering, with individuals reacting better and faster to stressful experiences such as exploring a new environment [11]. This could result in decreased neophobia that would allow, for example, to visit areas perceived as risky or approach and acquire novel food sources [12, 13]. Early discovery, examination and securing of resources could prove crucial for survival, particularly for invasive species that rely on novel and ephemeral food sources. For individuals of such species it is conceivable that covering ground rapidly and having short latencies to forage and drink, i.e. the characteristics of a fast exploration, could prove advantageous in a novel environment [14–16]. Exploring with a conspecific could allow to spend less time alert without sacrificing cautiousness, as alert time can be split between companions. Moreover, some species strongly rely on social cues to detect clumped food sources in an unpredictable habitat [17, 18]; when different and often novel food sources are available, such as during a colonisation event, a group can allow greater flexibility (coping faster with new situations) and better performances than individuals alone (see for example [19]). Apart from the conspecific presence in itself, attention has recently been given to the influence that the characteristics of the conspecifics have on the behaviour of a focal individual [3, 10]. Among other things, individuals were discovered to behave differently depending on their companions’ boldness [20], kinship [21], and social dominance [22]. Some aggressive or dominant individuals could for example be perceived as a stressor for their group-mates, thus increasing alert time and neophobia in difficult situations [23, 24], while other conspecifics could have the very opposite effect, decreasing neophobia and alert time [13]. This underlines a system of conspecific recognition and flexibility in behavioural responses that may be affected by differences in behavioural traits and experiences [25, 26].

The phenotype of the companion is not the only aspect that could influence an individual’s behaviour in a social context. One distinction to be made between conspecifics is if they are familiar or unfamiliar with one another, namely if they have learnt to recognize group mates with which they had repeated interactions or not [27–29]. The behavioural response to the presence of a familiar conspecific can be different from the response to the presence of an unfamiliar conspecific [30]. Firstly, antagonistic interactions are often less common among familiar than among unfamiliar conspecifics as the unfamiliar conspecifics may use such interactions to establish a new social dominance hierarchy [31]. Secondly, since animals living in social groups are prone to competition and other within-group stressors, familiarity between group-members has been argued to be an important factor keeping groups together, avoiding a continuous fission-fusion process that could be costly in the long run [28, 29, 31, 32]. Thirdly, an unfamiliar conspecific could represent an unknown risk and source of stress until the potential threats it presents are fully assessed [33]. Lastly, immediately trusting an unfamiliar individual from the first encounter could prove maladaptive, since an unfamiliar conspecific could exploit the newly formed connection without giving anything in return [34]. In this scenario, the unfamiliar individual would simply not be trustworthy enough to be used as a reliable source of vigilance or social information, and thus either be ignored or mistrusted [35–37].

The effects of conspecifics familiarity on animal behavioural responses, i.e. the difference in behavioural responses due to conspecific presence being either familiar or unfamiliar, has been mostly studied in fish [30], where it has been found to facilitate social learning, decrease stress and reduce aggression in social groups [30, 38, 39]. In the few studies available on birds it was shown that couples consisting of birds that had been familiar with each other for a long time were sometimes found to have higher fitness, possibly due to greater coordination and cooperation ([40–42], but see [43]). Other studies focused instead on the difference between familiar conspecific presence and conspecific absence [44]. In this study we extended the comparison to unfamiliar conspecifics, focusing on the effect of conspecific familiarity which, to our knowledge, has never been studied in relation to exploratory behaviour in birds.

Therefore, we argue that behavioural responses such as latency to forage and visit the ground in a novel environment, or time spent foraging and fraction of the novel environment visited, could be influenced by i) presence or absence of conspecific individuals; ii) relationship between conspecific individuals, i.e. if they are familiar or unfamiliar with one another. We argue that the presence of any conspecific could act by itself as a social buffer, lowering neophobic behaviours and resulting, for example, in shorter latencies to forage and more explored areas. However, as assessing the potential threats that an unknown conspecific provides could take time and be potentially stressful, it is possible that an unfamiliar conspecific could not be an effective social buffer. In contrast, a known companion would be a familiar feature in an unfamiliar situation: being alongside it during novel environment exploration could reduce neophobia, which would be particularly useful for a species with a rapid-expanding range or unpredictable habitat, as it would encounter many novel resources, stressors and social environments [45].

To address these questions we decided to use the house sparrow (*Passer domesticus*), as it is an opportunistic human commensal, and thus a species that often depends on clumped, novel and ephemeral food sources. House sparrows have been studied for processes of urbanization [46], dispersal [47] and range expansion [14, 45], as it is an invasive species in many areas of the world. Moreover, it is a highly sociable species, which has already been shown to use its social environment to obtain clues about unknown food sources, with individuals leading the foraging bout actively emitting assembly calls to their companions [18, 19]. For these reasons – being both highly sociable and an opportunistic invader – the house sparrow constitutes an ideal model species to examine the role of social environment in relation to exploratory efficiency (i.e., during novel environment exploration). During winter house sparrows reunite in mixed-sex flocks and often forage in small sub-flocks in urban areas. In this period of the year their social life is thus characterized by continuous fission-fusion dynamics, that allow them to come in contact with both familiar and unfamiliar individuals [48]. Moreover, while generally sedentary, first-year birds (like the ones that we used in our experiment) undergo extensive dispersal [49], while changes in local condition can force them to colonize new areas alongside human settlements [50, 51]. It is not uncommon for them to separate in same-sex couples or small groups, or even move alone for short periods of time ([52, 53], Authors unpublished observations).

We tested the exploratory behaviour of first-year house sparrows in an indoor aviary in three different social contexts: alone, in same-sex familiar pairs and in same-sex unfamiliar pairs. In the current study we did not test mixed-sex pairs. In the novel indoor aviary the sparrows could find food sources, water (on the ground), branches divided in ten sectors. We predicted that birds in the individual context (i.e. tested alone) would be the least bold, having longest latencies to exploit resources (i.e., forage at any food source for the first time) or visit potentially risky areas (i.e., the ground). They would also visit the fewest number of sectors in the novel environment. On the contrary, individuals in the familiar context would behave the most exploratory, having shortest latencies to forage and touch the ground and spending more time eating than when in the other contexts. Finally, individuals would explore differently when alongside an unfamiliar companion from when alongside a familiar one. The unfamiliar context could either cause a decrease of exploratory behaviour under the levels of individuals alone [54, 55], or result in an intermediate level of exploration, i.e. between the familiar and individual context [30].

## Methods

### Housing and study subjects

The study was conducted between March and June at the Konrad Lorenz Institute of Ethology (KLIVV, University of Veterinary Medicine) in Vienna, Austria (48°13’ N, 16°17’ E). All 96 house sparrows (48 males and 48 females) used in the experiment were born during the previous breeding season (252.46 ± 26.57 days of age at the beginning of the experiment. Measures reported here and henceforward are mean ± standard error of the mean) and reared by their parents in the same aviaries where they were born. We used only one-year-old birds to avoid age-related variations during tests [56, 57]. The birds were kept in mixed-sex outdoor enclosures (from now on “housing aviaries”), measuring 3.9 × 2 × 2.6 m (m) (l × w × h). Each housing aviary was equipped with a feeder (consisting of a metal bowl on a wooden pedestal, 1.2 m from the ground), small pine trees, which were usually used to roost, and four branches as additional perching places. Pine trees had the same size, shape and height (1.5 m) while branches came from trees near the research institute. All aviaries were provided with food (a mixture of millet, canary seeds, wheat, sunflower seeds, protein-based mash, plus apple slices and millet sprays hanging from the branches) and water poured in a dish on the ground [58, 59]. All the study subjects were housed together in 5 housing aviaries and all individuals not belonging to the age-class of the study subjects were removed from the 5 housing aviaries 50 days before the start of the experiment, leaving 19.2 ± 1.8 sparrows in each aviary (range: 15–25 sparrows). Sparrows from different aviaries had never been housed with each other (were completely unfamiliar with each other). Conversely, sparrows from the same housing aviary were either born in the same aviary or were kept together for at least 50 days before the start of the experiment (were thus familiar with each other). Different housing aviaries were located in four different corners of the Institute, and thus separated by trees and buildings and not in visual or acoustic contact. Two housing aviaries were in the same corner of the institute but were at the two extremities of a row of 12 aviaries, thus separated by ten other aviaries (25 m distant), all housing other birds unrelated to the experiment.

### Temporary housing and sub-flocks

The study subjects were further divided in 16 groups (8 groups of males and 8 groups of females) of 6 birds each: all groups were randomly composed of same-sex familiar individuals. There was no difference in body mass, wing length and tarsus length between groups of the same sex (data not shown). Two male groups and two female groups were then moved into 4 temporary aviaries, which were visually and acoustically isolated from the other temporary aviaries and only visually isolated from their own housing aviary. When all the birds in the first 4 groups had been tested once (7.08 ± 1.31 days), the birds were returned to their housing aviaries and the next 4 groups were moved to the temporary aviaries. The reasons for this transfer from housing to temporary aviaries were both practical and experimental. The temporary aviaries were not meant to stress the birds with novelties, and thus were similarly but more homogeneously equipped than the housing aviaries. Furthermore, the management, selection and capture of a bird inside a temporary aviary (containing only 6 birds) was much easier and less stressful than in the housing aviaries where more birds were housed together. A short food deprivation was also necessary for the experimental design and would have been difficult to achieve in the housing aviaries (see Exploration aviary and experimental protocol). Above all, a flock of six birds likely resulted in all birds in each temporary housing aviary closely interacting with one another. Hence, we considered the birds being familiar to one another for the purpose of the experiment. The temporary aviaries measured 3.7 × 1.9 × 2.5 m (l × w × h), and were equipped with a metal bowl on a pedestal (1.2 m from the ground), branches on the corners of the roof, one or two roosting trees (depending on the amount of roosting places provided) and a water dish on the ground. Features of these novel aviaries were, as far as possible, of the same size and in the same position in all four temporary aviaries. Birds in these aviaries were fed daily (in the morning) with ad libitum (roughly 300 g) standard mixture of seeds (wheat, canary seeds, sunflower seeds). To ensure that transferring the birds was not causing excessive stress all individuals were closely monitored after each relocation by observing them for a minimum of 3 h or until all birds drank and fed if that took longer. Moreover, we made sure that no bird was showing injuries or atypical behaviour, such as prolonged time spent on the floor or flying issues. The birds were left for two days to habituate to the temporary housing aviaries (enough time for captive house sparrows to habituate to a new environment; [17]) before testing began on the morning of the third day.

### Test order

We recorded the exploratory behaviour of all 96 individuals (48 males and 48 females), testing them in three social contexts, namely alone (individual context), with a familiar individual and with an unfamiliar same-sex individual. The total number of tests performed was 192: 96 individual tests, 48 familiar tests (each one with two individuals, 96 individuals tested) and 48 unfamiliar tests (each one with two individuals, 96 individuals tested) (Additional file 1: Figure S1). Thus, every bird was tested thrice, once in the familiar, once in the unfamiliar and once in the individual context, independently from all the other individuals considered. For each bird the order of the three tests was randomized across contexts: after all 96 birds had been tested in one context (32 of them in the individual context, 32 in the familiar and 32 in the unfamiliar context) we started two new rounds of tests where we tested each bird in the two remaining contexts. Each round had then 32 individual tests, 16 familiar tests (32 individuals tested) and 16 familiar tests (32 individuals tested). Successive tests of the same birds were separated by 37.24 ± 13.9 days, a period that is considered more than sufficient to avoid learning effects [20]. We did not return the birds to the housing aviaries until every bird in the 4 groups had been tested, in order to maintain the social groups consistent among tests. All tests were conducted between 2 h after sunrise and 1 h before sunset: the hour of the test was randomized between individuals and contexts.

At the end of the experiment half of the birds (48 individuals, 24 males and 24 females) were randomly chosen as “focal”. In each social test individuals were either focal or companion: no individual was both focal and companion (Additional file 1: Figure S1). No siblings were tested together.

### Exploration aviary and experimental protocol

Two hours prior to each test the food bowl was removed from the temporary aviaries of the individual(s) scheduled for the test, in order to normalize the foraging motivation [60]. After the birds undergoing testing were capture and removed from the temporary housing aviary, food was returned to the other individuals. Exploratory behaviour has long been studied via novel environment tests conducted in relatively small rooms [61], tents [45] or cages [54, 62, 63]. We decided to assess exploratory behaviour in an indoor novel environment (exploration aviary), which measured 8.3 × 8.7 × 2.5 m (l × w × h) and was equipped with a number of features to simulate a natural environment. Due to its size, novelty and thus possibly also perceived risk the exploration aviary required a longer time to be properly assessed by house sparrows. Thus, we decided to run 2-h long tests. Light was both natural, coming from the semi-transparent roof, and artificial (9 neon lights, always turned on); the floor was covered with wood shavings, as in all the outdoor aviaries. A quarter of the 72.21 square meters of the exploration aviary was covered by branches. There were also four food sources, of which 3 were sprays of millet hanging from the branches and one was a food bowl on a pedestal with a mixture of seeds and a spray of millet inside. Water was positioned on the ground, as in the housing and temporary aviaries. The branches and the other perching areas were differentiated in 10 sectors, corresponding to spatial locations independent from one another. We rarely observed birds hopping back and forth from different sectors, as moving from one to the other usually required at least a brief flight. All observations were done via a one-way see-through plastic mirror on the left wall of the exploration aviary. All tests were recorded using three webcams (LifeCam Studio, Microsoft. Article number: Q2F-00015 and Q2F-00016). Video data were processed through iSpy, a free open source software (version 6.3.0.0). The birds were also visually monitored by one of the authors (B.T.) through the one-way see-through plastic mirror previously mentioned. After carefully measuring every feature of the exploration aviary we reviewed all video footage to estimate total travel distance [64]. One fifth of the individuals were reviewed by both G.F. and B.T. to account for possible effects of subjectivity.

At the beginning of every test the study subjects were captured at the temporary aviaries with hand-nets as quickly as possible (usually less than 4 min), and then transferred via a small cloth bag to a two-parted cage (2 × 0.5 × 0.5 m (l × w × h)) inside the exploration aviary. All individuals were unable to see their companion in this cage, but they were able to see the exploration aviary. After 10 min of habituation, the cage was opened from outside the exploration aviary using a remote system. As soon as the cage was open the test started. Each test lasted 2 h, after which we captured the birds from the exploration aviary and we released them back to their temporary aviaries. For all individuals (both focal and companion) we recorded a number of variables, such as i) latency to forage; ii) latency to touch the ground; iii) number of sectors visited and iv) time spent foraging. The latter was defined as time spent by birds pecking at the food: any pause in the pecking longer than 3 s was recorded. Birds that did not eat or touch the ground were assigned a latency of 7201 s [65]. Through the analysis of the video footage for each test in the familiar and unfamiliar context we also recorded, for all conspecific pairs: v) number of aggressive interactions (i.e. biting and chasing); vi) number of following bouts. Following bouts were defined as the flights of both birds from one sector to another, taking off within 3 s of each other (similarly as in [17]).

### Ethical note

Capture, housing and handling of birds were in accordance with the relevant Austrian laws and were licensed by the government of Vienna (MA 22) license number 424/2011. The experiment reported in this study complies with current laws on animal experimentation in Austria and the European Union. This study was approved by the institutional ethics committee (University of Veterinary Medicine, Vienna) and the national authority according to 8ff (rules) of Law for Animal Experiments Tierversuchsgesetz - TVG, licence number GZ 68.205/0220-II/3b/2012.

The condition and health of experimental birds were monitored on a daily basis. No individual died during the 5-month long experiment.

### Statistical analyses

All data were analysed using R version 3.2.1 [66]. All statistical tests were two-tailed. The significance threshold was set at *α* = 0.05. Exploratory behaviour in a novel environment was analysed using Generalized Linear Mixed Models (GLMMs). GLMMs are often used wherever data are non-normally distributed and random effects possibly account for part of the variance. The models were fitted using the ‘glmer’ function within the package ‘lme4’ (1.0.5) for R version 3.2.1 [67]. Each dependent variable was analysed using a separate model. Sex, context (alone, i.e. individual context, with a familiar conspecific, with an unfamiliar conspecific) and their interaction were fitted as categorical fixed effects. We also added test order (i.e. if the test took place during the first, second or third round of tests) and part of the day when the test took place (i.e. morning or afternoon) as fixed effects. The dependent variables relative to each individual were i) fraction of sectors visited (out of a maximum of 10); ii) latency to forage (seconds); iii) latency to touch the ground (seconds). We analysed the fraction of sectors visited using logistic regression for proportion (logit link), while the latency to forage and the latency to touch the ground were modelled with gamma distribution (log link). The log link was chosen because the use of the canonical (inverse) link often caused models to fail to converge. The gamma models that did converge with the inverse link had similar results to the ones with the log link. Total distance travelled was correlated with number of sectors visited, and time spent foraging was correlated with latency to forage (see Results); analysis of both these variables are shown in Additional file 1: Table S1 and S2. We also analysed a variable that was not related to individuals alone, but to each pair, i.e. iv) number of following bouts in each hour of test. In order to focus on differences in this behavioural response between the first and the second hour of each test we used as dependent variable the number of following bouts relative to each of the two hours that comprised a test. Thus, for this dependent variable we also fitted as categorical fixed effect ‘hour’, i.e. if the number of following bouts corresponded to the first or second hour of experimental observation. As the two hours of the same test could not have been considered independent we added a random factor ‘test’. We analysed this variable using Gamma distribution (log link). Aggressive interactions could not be analysed as their number was too low to be informative (see Results). We also tested for correlation using the ‘Kendall’ package [68] applying a false discovery rate correction.

Estimates and significance of the fixed effects were obtained using the ‘Anova’ function within the ‘car’ package [69], while the ‘confint.merMod’ function within the ‘lme4 package was used to obtain intervals of confidence. To differentiate among three or more groups we performed post-hoc analyses of contrasts with the ‘lsmeans’ function within package ‘lsmeans’ [70] applying the Tukey method adjusted for multiple comparisons. Results were back-transformed and compared to those obtained with ‘glht’ in the ‘multcomp’ package [71], to which they were very similar. We entered as random effects the identity of the bird (as every bird participated once in all three tests).

Social context was also entered as a repeated measure to account for the non-independence of birds’ behavioural response to each context [63].

## Results

### Latency to touch the ground

House sparrows in the individual context had longest latency to touch the ground. The main effect ‘social context’ had a significant influence on the latency to touch the ground (df = 2, χ^2^ = 22.380, *p* < 0.0001). The social context × sex interaction was also significant (df = 2, χ^2^ = 8.751, *p* = 0.013). However, the main effect ‘sex’ was not significant (df = 1, χ^2^ = 0.191, *p* = 0.663), even if females in the unfamiliar context had longer latency to touch the ground than males in the unfamiliar context (Table 1).

**Table 1:** Effect of ‘part of the day’ (morning or afternoon), ‘round of tests’ (first, second or third), ‘sex’ (female or male), ‘social context’ (individual, unfamiliar, familiar) and interaction between ‘social context’ and ‘sex’ on latency to touch the ground

Fixed effect	Comparison	Estimate	2% CI	98% CI	*P* value
Part of the day	Morning vs afternoon	− 0.183	**− 0.290**	**− 0.076**	**0.0004**
Sex	Female vs male	0.051	−0.185	0.287	0.6581
Round	First vs second	0.160	**0.045**	**0.276**	**0.0021**
	First vs third	0.200	**0.085**	**0.315**	**0.0001**
	Second vs third	0.039	0.075	−0.154	0.6817
Social context	Individual vs unfamiliar	−0.114	− 0.228	0.0006	0.041
	Individual vs familiar	− 0.224	**−0.338**	**− 0.109**	**<.0001**
	Familiar vs unfamiliar	−0.110	−0.224	0.004	0.050
Social context × sex	Female: individual vs unfamiliar	0.024	−0.136	0.185	0.9283
	Female: individual vs familiar	−0.170	**−0.330**	**− 0.009**	**0.0283**
	Female: familiar vs unfamiliar	0.194	**0.031**	**0.356**	**0.0101**
	Male: individual vs unfamiliar	−0.252	**−0.413**	**− 0.089**	**0.0005**
	Male: individual vs familiar	−0.278	**−0.440**	**− 0.116**	**0.0001**
	Male: familiar vs unfamiliar	0.026	−0.135	0.188	0.9163
Sex × social context	Individual: female vs male	0.077	−0.183	0.338	0.815
	Unfamiliar: female vs male	−0.199	**−0.460**	**0.063**	**0.009**
	Familiar: female vs male	−0.031	−0.292	0.230	0.534
Random effect		**Variance**	**± SE**		
Individual identity		0.090	± 0.303		

Coefficients and 96% confidence intervals are presented; statistically significant comparisons (zero is not included in the interval) are in **bold**. *P* values obtained with Tukey method adjusted for multiple comparisons. Results are in the log (not in the response) scale. ‘Individual identity’ is fitted as random effect; variance associated with it is shown

**Fig. 1:**
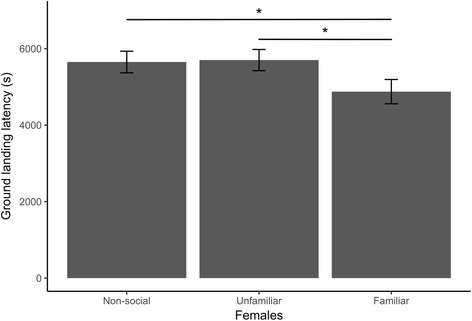
Social context influence on ground landing latency in female house sparrows in a novel environment. Females exploring with a familiar companion had significantly shorter latencies to land on the ground than females exploring with an unfamiliar companion or alone. Means and standard error of the mean are shown. *P < 0.05

**Fig. 2:**
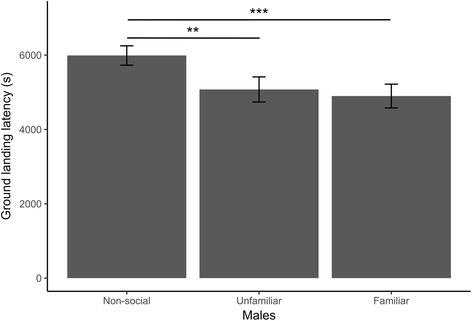
Social context influence on ground landing latency in male house sparrows in a novel environment. Males exploring alone had significantly longer latencies to land on the ground than males exploring with an unfamiliar or a familiar companion. Means and standard error of the mean are shown. ***P < 0.001. **P < 0.01. *P < 0.05

Females touched the ground earliest in the familiar context, significantly sooner than in both the individual and unfamiliar context (Table 1, Fig. 1). Males did not differ between the unfamiliar and familiar contexts but touched the ground last in the individual context, significantly later than in both the unfamiliar and familiar context (Table 1, Fig. 2).

The main effect ‘test order’ had a significant influence on the latency to touch the ground (df = 2, χ^2^ = 19.998, *p* < 0.0001). Individuals visiting the room for the second and third time touched the ground sooner than when at the first experience, but they did not differ in their latency to touch the ground between the second and third tests (Table 1). Finally, the main effect ‘time of day’ significantly influenced the latency to touch the ground (df = 1, χ^2^ = 12.323, *p* = 0.0005), with individuals touching the ground sooner in the afternoon than in the morning (Table 1).

### Latency to forage

House sparrows in the individual context had longest latency to forage. The main effects of ‘social context’ was significant (df = 2, χ^2^ = 24.109, *p* < 0.0001) and so was the social context × sex interaction (df = 2, χ^2^ = 7.319, *p* = 0.026): males had longer foraging latency in the individual context than both in the unfamiliar and familiar context (Table 2). Females had shorter foraging latency in the familiar than in the individual context but did not differ between unfamiliar and individual context and between unfamiliar and familiar context (Table 2). In contrast to males, female foraging latency in the unfamiliar context was intermediate between the individual and familiar contexts though the difference was not significant. Accordingly, there was also a significant sex difference limited to the unfamiliar context, with males having significantly shorter latency to forage than females (Table 2). The main effect of ‘sex’ was not significant (df = 1, χ^2^ = 0.168, *p* = 0.682). The main effect ‘test order’ had a marginally significant influence on the latency to forage (df = 2, χ^2^ = 5.992, *p* = 0.050), with sparrows foraging marginally sooner in the third round of tests (Table 2).

**Table 2:** Effect of ‘part of the day’ (morning or afternoon), ‘round of tests’ (first, second or third), ‘sex’ (female or male), ‘social context’ (individual, unfamiliar, familiar) and interaction between ‘social context’ and ‘sex’ on latency to forage

Fixed effect	Comparison	Estimate	2% CI	98% CI	*P* value
Part of the day	Morning vs afternoon	−0.125	− 0.431	0.181	0.403
Sex	Female vs male	−0.264	− 0.667	0.137	0.177
Round	First vs second	0.010	−0.358	0.377	0.998
	First vs third	−0.318	− 0.686	0.049	0.088
	Second vs third	−0.328	−0.700	0.044	0.082
Social context	Individual vs unfamiliar	−0.711	**−1.075**	**− 0.348**	**0.0001**
	Individual vs familiar	−0.614	**−0.974**	**− 0.253**	**0.0001**
	Familiar vs unfamiliar	0.098	−0.270	0.465	0.534
Sex × social context	Individual: female vs male	0.060	−0.467	0.587	0.815
	Unfamiliar: female vs male	−0.692	**−1.234**	**− 0.149**	**0.009**
	Familiar: female vs male	−0.162	− 0.698	0.374	0.534
Social context × sex	Female: individual vs unfamiliar	−0.336	− 0.842	0.171	0.241
	Female: individual vs familiar	−0.503	**−1.007**	**−0.002**	**0.041**
	Female: familiar vs unfamiliar	−0.167	−0.687	0.354	0.716
	Male: individual vs unfamiliar	−1.087	**−1.608**	**−0.567**	**<.0001**
	Male: individual vs familiar	−0.725	**−1.236**	**−0.214**	**0.0016**
	Male: familiar vs unfamiliar	0.362	−0.155	0.880	0.205
Random effect		**Variance**	**± SE**		
Individual identity		0.495	± 0.703		

Coefficients and 96% confidence intervals are presented; statistically significant comparisons (zero is not included in the interval) are in **bold**. *P* values obtained with Tukey method adjusted for multiple comparisons. Results are in the log (not in the response) scale. ‘Individual identity’ is fitted as random effect; we show the variance associated with it

### Number of sectors visited

House sparrows in the individual context visited the least sectors. The main effect of ‘social context’ was significant (df = 2, χ^2^ = 10.481, *p* = 0.005): birds in the individual context visited less sectors than birds in both unfamiliar and familiar contexts (Table 3). The main effect of ‘sex’ showed a non-significant tendency (df = 1, χ^2^ = 3.279, *p* = 0.070) and the greatest difference being in the unfamiliar context (Table 3). The interaction between the two factors was not significant; however, we kept it in the model as it made theoretical sense in the context of our question. The main effect ‘test order’ had a significant influence on the number of sectors visited (df = 2, χ^2^ = 42.796, *p* < 0.0001, with individuals visiting less sectors with increasing test order, i.e. visiting the most sectors when they first entered the room and the least the third time. Finally, individuals visited more sectors in the morning than in the afternoon (df = 1, χ^2^ = 9.849, *p* < 0.002, Table 3).

**Table 3:** Effect of ‘part of the day’ (morning or afternoon), ‘round of tests’ (first, second or third), ‘sex’ (female or male), ‘social context’ (individual, unfamiliar, familiar) and interaction between ‘social context’ and ‘sex’ on number of sectors visited

Fixed effect	Comparison	Estimate	2% CI	98% CI	*P* value
Part of the day	Morning vs afternoon	−0.328	**−0.542**	**−0.113**	**0.002**
Sex	Female vs male	0.313	−0.040	0.665	0.069
Round	First vs second	−0.370	**−0.609**	**− 0.130**	**0.005**
	First vs third	−0.642	**−0.882**	**− 0.403**	**<.0001**
	Second vs third	−0.272	**−0.512**	**− 0.032**	**0.0161**
Social context	Individual vs unfamiliar	0.284	**0.044**	**0.524**	**0.011**
	Individual vs familiar	0.268	**0.031**	**0.506**	**0.017**
	Familiar vs unfamiliar	−0.016	−0.255	0.222	0.985
Sex × social context	Individual: female vs male	0.286	−0.136	0.708	0.165
	Unfamiliar: female vs male	0.455	**0.031**	**0.880**	**0.028**
	Familiar: female vs male	0.197	−0.224	0.618	0.337
Social context × sex	Female: individual vs unfamiliar	0.199	−0.138	0.536	0.3215
	Female: individual vs familiar	0.313	−0.024	0.649	0.0621
	Female: familiar vs unfamiliar	0.113	−0.223	0.450	0.6917
	Male: individual vs unfamiliar	0.369	**0.028**	**0.709**	**0.023**
	Male: individual vs familiar	0.224	−0.111	0.558	0.236
	Male: familiar vs unfamiliar	−0.145	−0.483	0.193	0.549
Random effect		**Variance**	**± SE**		
Individual identity		0.552	± 0.743		

Coefficients and 96% confidence intervals are presented; statistically significant comparisons (zero is not included in the interval) are in **bold**. *P* values obtained with Tukey method adjusted for multiple comparisons. Results are in the log (not in the response) scale. ‘Individual identity’ is fitted as random effect; variance associated with it is shown

### Social behaviour variables

The number of following bouts recorded during the entire duration of the test (2 h) was influenced by the familiarity of the pair, with familiar pairs performing more following bouts than unfamiliar pairs (df = 1, χ^2^ = 4.619, *p* = 0.032). Sparrows performed more following bouts during the second hour in both contexts (df = 1, χ^2^ = 5.964, *p* = 0.015); however, the difference was much more pronounced in the unfamiliar context. Accordingly, the treatment × hour interaction was significant (df = 1, χ^2^ = 4.905 *p* = 0.027): sparrows in the familiar context performed significantly more following bouts than those in the unfamiliar context, but only in the first hour (Table 4). The ‘social context’ × sex interaction was not significant and was excluded from the model. The main effect ‘sex’ was also not significant (df = 1, χ^2^ = 0.024, *p* = 0.877). The main effect ‘test order’ was significant (df = 2, χ^2^ = 9.174, *p* = 0.010), with birds in the first round performing more following bouts than in the third (Table 4). The total number of aggressive interactions recorded was very low, as we recorded 44 aggressive interactions in 48 tests in the unfamiliar context and 36 aggressive interactions in 48 tests of familiar context (0.42 aggressive interaction per hour).

**Table 4:** Effect of ‘part of the day’ (morning or afternoon), ‘round of tests’ (first, second or third), ‘sex’ (female or male), ‘social context’ (individual, unfamiliar, familiar), ‘hour’ (first or second hour of the test) and interaction between ‘social context’ and ‘hour’ on number of following bouts recorded in one hour

Fixed effect	Comparison	Estimate	2% CI	98% CI	*P* value
Part of the day	Morning vs afternoon	0.043	−0.029	0.116	0.221
Sex	Female vs male	0.007	−0.085	0.098	0.877
Hour	Second vs first	−0.063	**−0.089**	**− 0.037**	**<.0001**
Round	First vs second	0.060	−0.033	0.154	0.259
	First vs third	0.121	**0.024**	**0.219**	**0.007**
	Second vs third	0.061	−0.040	0.162	0.307
Social context	Unfamiliar vs familiar	0.059	**0.001**	**0.121**	**0.045**
Social context × hour	First hour: unfamiliar vs familiar	0.074	**0.003**	**0.144**	**0.032**
	Second hour: unfamiliar vs familiar	0.018	−0.045	0.081	0.554
Hour × treatment	Familiar: second hour vs first hour	−0.035	**−0.064**	**− 0.006**	**0.014**
	Unfamiliar: second hour vs first hour	−0.091	**−0.134**	**− 0.048**	**<.0001**
Random effect		**Variance**	**± SE**		
Individual identity		0.008	± 0.087		
Test		0.007	± 0.085		

Coefficients and 96% confidence intervals are presented; statistically significant comparisons (zero is not included in the interval) are in **bold**. *P* values obtained with Tukey method adjusted for multiple comparisons. Results are in the log (not in the response) scale. ‘Individual identity’ and ‘test’ are fitted as random effects; variances associated with them are shown

### Correlation between the dependents variables

Total distance travelled was highly correlated with sectors visited (Kendall Rank Correlation, tau = 0.668, *p* < 0.001). Such correlation was strongest in unfamiliar (tau = 0.700, p < 0.001) and familiar (tau = 0.681, *p* < 0.001) contexts: in individual context however it was much weaker and not significant after correction. Time spent foraging was negatively correlated with foraging latency (tau = − 0.423, p < 0.001). Ground latency was weakly but significantly negatively correlated with fraction of sectors visited (tau = − 0.298, *p* < 0.001): this correlation was stronger when considering only the individual (tau = − 0.452, *p* < 0.001) or the unfamiliar context (tau = − 0.434, *p* < 0.001), but very weak when considering the familiar one (tau = − 0.184, *p* = 0.16). A full correlation matrix is provided in the (Additional file 1: Table S3).

## Discussion

Our experiment analysed numerous variables in three contexts for both sexes and provided various results. We provide a short summary of the most relevant results below.

1. Both sexes in the individual context had longer latency to land on the ground than in the familiar context. Also, house sparrows visited less sectors in the individual context than either in the familiar or unfamiliar contexts when averaging across sexes.
2. Familiar pairs performed more following bouts than unfamiliar pairs, with the difference being more pronounced in the first hour of testing and non-significant in the second hour.
3. Females landed on the ground sooner when in the familiar context than either when in the unfamiliar or individual context. They also foraged sooner in the familiar than in the individual context. Females behavioural responses did not differ significantly between the individual and the unfamiliar contexts. Males’ behavioural responses, on the other hand, significantly differed between the unfamiliar and the individual contexts (they foraged and landed on the ground sooner, spent more time foraging and visited more sectors when coupled with a companion); latency to forage and to go to the ground also differed between familiar and individual contexts.
4. When considering only the unfamiliar context males foraged sooner (and thus for longer, Additional file 1: Table S1) than females. Males in general visited also marginally more sectors than females, with this difference being more pronounced in the unfamiliar context.
5. ‘Test order’ had an effect on every dependent variable while ‘part of the day’ affected ground latency and number of sectors visited.

The first result is consistent with the social facilitation effect on exploratory behaviour [9, 12, 13]. Interestingly, we did not find any difference between the total distance travelled between sparrows moving in pairs and in the individual context (Additional file 1: Table S2). As sparrows in pairs visited more sectors, we can assume that they covered more ground even if travelling the same distance than when in the individual context. We may thus speculate that even if the presence of a companion increases the number of sectors visited, it could possibly have no effect on the energy spent in movement, as the same distance would still be travelled in the individual context, even if perhaps in a more restricted area. However, there was also a strong correlation between sectors visited and distance travelled, meaning that across all three contexts there is a relationship between the two measures.

The second result, the difference in the number of following bouts between familiar and unfamiliar pairs is interesting for three reasons. Firstly, as the difference was highly significant when confronting the first hour and non-significant when confronting the second, we could be seeing a quick process of habituation to the unfamiliar conspecific [31]. Both contexts performed more following bouts in the second hour of the test – as it is to be expected, as they gain confidence with the environment and start foraging and going to the ground: this suggests that familiarity in this species could have an influence on behavioural responses only on a relatively restricted temporal scale. Secondly, this result offers an insight in how two birds move together in a novel environment: following one another from one sector to the other might be the cause of social facilitation, i.e. increase in the number of sectors visited. Lastly, the higher number of following bouts in familiar pairs could be a clue on how familiarity influences behavioural responses: being used to move with another individual would be, for example, the reason for quicker coordination in case of attack or discovery of a food source [72]. We encourage future studies to investigate how differences in the behavioural responses of familiar and unfamiliar pairs fade after a certain amount of time. Also, it is still unclear if pairs or groups of birds move differently according to their familiarity when in a novel environment.

The third result underscores how the behavioural response to unfamiliar individuals differed depending on the sex of the individuals. Females in the familiar context had significantly shorter latency to visit the ground than when in the unfamiliar context. This result is in line with previous studies performed in fish where familiarity was associated with increased time spent exploring a novel object, latency to emerge from a refuge and faster habituation to a novel environment [30, 73, 74]. The latency to visit the ground is particularly important, as birds usually perceive the soil as a higher-risk area compared to perches that were higher off the ground [20, 60], and in our set-up the birds rarely visited the ground for reasons different than going to a water source [BT unpublished observation]. For these reasons we argue that a shorter latency to venture on the ground provides a strong indication of reduced perceived predation risk. It is worth noting that this result could also have been due to distraction due to a higher frequency of aggressive interactions in the unfamiliar context. However, the total number of aggressive interactions was very small and thus unlikely to have a significant effect on other behavioural traits.

Males did not differ in their behaviour between the unfamiliar and familiar contexts. The behavioural responses either differed from between the individual context and whenever they moved with a companion, independently of its familiarity (latencies to touch the ground and forage) or differed only between the individual and the unfamiliar context (sectors visited, time foraging). Conversely, females significantly decreased their latencies to forage and to touch the ground (and slightly increased the number of sectors visited, even if not significantly) only when released alongside a familiar group-mate.

Hence the fourth result: males exploring with an unfamiliar companion visited more sectors, spent more time eating and started foraging sooner than females with an unfamiliar companion. Only in recent years has the role of sex been taken into consideration in familiarity studies [29, 75, 76]. In a parallel work on Mediterranean killifish (*Aphanius fasciatus*) it was found that in exploring same-sex pairs only females showed reduced latency to emerge from a refuge if their companion was familiar instead than unfamiliar, whereas males showed the opposite trend [73]. Moreover, a study on brown-headed cowbirds (*Molothrus ater*) found that females spent more time interacting with familiar conspecifics than unfamiliar conspecifics [29], which is consistent with our current results. There is thus a growing body of evidence suggesting that females of different taxa value the familiarity of conspecific individuals differently than males, with our findings strengthening this hypothesis.

Differences in how the two sexes approach unfamiliar conspecifics could have a number of non-exclusive explanations. A different response to novel environments between females and males has already been shown in some previous studies [20, 54]. The lack of prior interactions between two unfamiliar females could have left them with very limited information about each other’s reliability as a source of vigilance [34–36]. In this case, unfamiliar females could have failed to reduce the anti-predation alertness of the other conspecific because they did not consider each other a reliable source of information. On the other hand, male house sparrows have been shown to be quicker than females to habituate to a potential disturbance (i.e. human disturbance near an unfamiliar object) [77], and less risk-averse than females [78]. This could also be the case of our study, as there is the chance that males could have habituated to the new companion quicker, without giving importance to previous experiences with it.

There are also a number of potential functional explanations. As house sparrow males are the ones picking and defending the nest site it would be paramount for them to assess and utilize the resources of a novel area as quickly as possible; even if this means exposing themselves to risks, such as novel predators or stressors [79]. In house sparrows, females were found to follow their companions to food sources, while males on the contrary were more consistently followed [80]. Because of this, females would have an advantage in carefully evaluating their companions, since they would depend more on the social information they provide.

Another possible explanation for our results would be that females value familiarity with their flock-mates because it could lead to help (decreased harassment, conjunct mobbing, shared alarmed behaviour) especially during the semi-colonial breeding season. In particular, it was recently shown that female cowbirds that preferred familiar connections laid more eggs during the breeding season [75]. Social instability can be costly due to increased rate of aggression, higher stress and lower reproductive output [75, 81] and in particular, stronger social bonds between females may lead to higher fitness compared to conspecifics with weaker social bonds, as shown in social mammals [75, 82, 83]. Moreover, birds are more likely to mob possible predators with familiar conspecifics than with unfamiliar conspecifics [36].

Finally, our work shows that, depending on the sex of the individuals, a familiar companion can strongly influence exploration in a social passerine bird, a situation particularly important for invasive and range-expanding species, such as the house sparrow and the brown-headed cowbird. Exploring a new area can indeed result in the chance of encountering unfamiliar conspecifics and in such a situation it would be important to fine-tune behavioural responses between familiar individuals and newly met strangers. The tendency to behave differently according to conspecific familiarity could prove to have a key role in the social environment structure, possibly as a factor keeping groups cohesive when exploring new territories [22, 75]. We may speculate that females behaving differently according to conspecific familiarity may be a factor in the social structure of sociable passerine bird flocks [75, 76], and maybe also of other different taxa [72, 73].

The test order had a strong influence on the house sparrow behavioural response to the novel environment. In particular, individuals had shortest ground latencies during the first round of tests, and significantly longer ground latencies in the second and third test – which did not differ between them. The pattern was the same also for total distance travelled (shortest in the first round of tests, Additional file 1: Table S2) and time spent foraging (longest time spent foraging in the first round of tests, Additional file 1: Table S1). The number of sectors visited was not only greater during the first round of tests with respect to both the others, but the second round of tests also saw a fewer number of sectors visited with respect to the third. Thus, all behavioural responses showed a slower, less extensive exploration after the very first round of tests [84] which has been known to happen also for house sparrows [77]. We cannot completely exclude the possibility that the effect was not due to habituation to the experimental aviary, but to the progress of the season [85]. However, in that case we would have possibly seen a greater difference in behavioural responses not between the first round of tests and the other two, but between the second and the third round of tests due to the onset of the breeding season.

A possible limit of our study was that we did not control for acute physiological stress responses, as all birds after being rapidly captured had only 10 min in the habituation cage. Future studies could definitely try to integrate stress responses analysis when investigating the effect that familiar and unfamiliar conspecifics have on the focal birds. Also, we encourage future studies to address the reasons behind this sex difference in the response to familiarity, for example by seeing if more exploratory males can be more or less attractive to females [86]. Moreover, it could be interesting to test how the fission-fusion structure of winter flocks of house sparrows could vary according to the sex of the individuals, and verify if females are more nuclear to the subgroups than males.

## Conclusions

We found evidence that pairs of familiar female house sparrows released in a novel environment landed faster on the ground than both in the unfamiliar and individual contexts. Males on the other hand did not differ in their behavioural responses between unfamiliar and familiar contexts, but had shorter latencies to land and forage, ate more and visited more sectors when in the unfamiliar context than in the individual one. Bird species are an important model in the field of exploratory behaviour, which nonetheless has been rarely considered in relation to the social environment. We provided evidence of the complex effects of social context on novel environment exploration. In particular, to the best of our knowledge this is the first evidence of the effect of conspecific familiarity on a behavioural response during novel environment exploration in birds: for the first time we tried to determine the effects of unfamiliar conspecifics alongside the usual comparison between familiar conspecifics and no conspecifics. Differences in the social context (i.e. alone, with an unfamiliar or with a familiar conspecific) impacted how both sexes exploited resources in a novel environment, an effect possibly paramount for invasive and opportunistic species.

## Additional file

Additional file 1: **Table S1.** Output of LMM with ‘time spent foraging’ as dependent variable. Effect of ‘part of the day’ (morning or afternoon), ‘round of tests’ (first, second or third), ‘sex’ (female or male), ‘social context’ (individual, unfamiliar, familiar) and interaction between social context and sex on time spent foraging. Fixed effect with significance obtained with ‘car’ package are presented. Coefficients and 96% confidence intervals are presented; statistically significant comparisons (zero is not included in the interval) are in bold P values obtained with Tukey method adjusted for multiple comparisons. **Table S2.** Output of GLMM with ‘total distance travelled’ as dependent variable (family Gamma, link = log). Effect of ‘part of the day’ (morning or afternoon), ‘round of tests’ (first, second or third), ‘sex’ (female or male), ‘social context’ (individual, unfamiliar, familiar) on total distance travelled. Interaction between social context and sex was excluded as not significant. Fixed effect with significance obtained with ‘car’ package are presented. Coefficients and 96% confidence intervals are presented; statistically significant comparisons (zero is not included in the interval) are in bold. *P* values obtained with Tukey method adjusted for multiple comparisons. **Table S3.** Correlation matrix between all dependent variables. Tau values obtained through Kendall Rank correlation. Results in bold are significant. False discovery rate correction was applied to value of α. **Figure S1.** An example of our test sorting. Boxes with the same colour (either red or blue) represent sparrows from the same aviary (familiar with each other). Each curved double arrow is a familiar context test, each straight double arrow is an unfamiliar context test, each point is an individual context test. Colours of arrows/points represent the test round: green first round of tests, yellow second round of tests, black third round of tests. (DOCX 139 kb)
