# Supplementary material for: House sparrows’ (Passer domesticus) behaviour in a novel environment is modulated by social context and familiarity in a sex-specific manner
Source: Front Zool. 2018 Apr 20;15:16. doi: 10.1186/s12983-018-0267-8 (PMC5910580; doi:10.1186/s12983-018-0267-8)
Supplement: Supplementary file 1 — Table S1. Output of LMM with ‘time spent foraging’ as dependent variable. Effect of ‘part of the day’ (morning or afternoon), ‘round of tests’ (first, second or third), ‘sex’ (female or male), ‘social context’ (individual, unfamiliar, familiar) and interaction between social context and sex on time spent foraging. Fixed effect with significance obtained with ‘car’ package are presented. Coefficients and 96% confidence intervals are presented; statistically significant comparisons (zero is not included in the interval) are in bold P values obtained with Tukey method adjusted for multiple comparisons. Table S2. Output of GLMM with ‘total distance travelled’ as dependent variable (family Gamma, link = log). Effect of ‘part of the day’ (morning or afternoon), ‘round of tests’ (first, second or third), ‘sex’ (female or male), ‘social context’ (individual, unfamiliar, familiar) on total distance travelled. Interaction between social context and sex was excluded as not significant. Fixed effect with significance obtained with ‘car’ package are presented. Coefficients and 96% confidence intervals are presented; statistically significant comparisons (zero is not included in the interval) are in bold. P values obtained with Tukey method adjusted for multiple comparisons. Table S3. Correlation matrix between all dependent variables. Tau values obtained through Kendall Rank correlation. Results in bold are significant. False discovery rate correction was applied to value of α. Figure S1. An example of our test sorting. Boxes with the same colour (either red or blue) represent sparrows from the same aviary (familiar with each other). Each curved double arrow is a familiar context test, each straight double arrow is an unfamiliar context test, each point is an individual context test. Colours of arrows/points represent the test round: green first round of tests, yellow second round of tests, black third round of tests. (DOCX 139 kb) [file 12983_2018_267_MOESM1_ESM.docx]

**SUPPLEMENTARY MATERIAL**

**Sex difference in the role of familiarity during novel environment exploration in an urban species**

Beniamino Tuliozi^1^, Gerardo Fracasso^1,2^, Herbert Hoi^3^, Matteo Griggio^1, *^

^1^ Department of Biology, University of Padova, Via U. Bassi 58/B, I-35131 Padova, Italy.

^2^ Evolutionary Ecology Group, Department of Biology, University of Antwerp, Universiteitsplein 1, B-2610 Wilrijk, Belgium.

^3^ Konrad Lorenz Institute of Ethology, Department of Integrative Biology and Evolution, University of Veterinary Medicine Vienna, Savoyenstrasse 1a A-1160, Austria.

* Corresponding author: Matteo Griggio, E-mail: matteo.griggio@unipd.it.

**Table s1**. Output of LMM with ‘time spent foraging’ as dependent variable. Effect of ‘part of the day’ (morning or afternoon), ‘round of tests’ (first, second or third), ‘sex’ (female or male), ‘social context’ (individual, unfamiliar, familiar) and interaction between social context and sex on time spent foraging. Fixed effect with significance obtained with ‘Anova’ function in ‘car’ package are presented. Coefficients and 96% confidence intervals are presented; statistically significant comparisons (zero is not included in the interval) are in **bold**. P values obtained with Tukey method adjusted for multiple comparisons.

| **Fixed effect** | **Comparison** | **Estimate** | **2% CI** | **98% CI** | **P value** |
| --- | --- | --- | --- | --- | --- |
| Part of the day  df = 1, χ^2^ = 1.200, p = 0.273 | Morning vs afternoon | 32.47 | -28.408 | 93.339 | 0.2743 |
| Sex  df = 1, χ^2^ = 0.216, p = 0.642 | Female vs male | 18.562 | -64.566 | 101.690 | 0.643 |
| Round  df = 2, χ^2^ = 21.466, p < 0.0001 | First vs second | -78.73 | **-131.345** | **-15.014** | **0.0241** |
|  | First vs third | -136.90 | **-209.617** | **-64.177** | **<.0001** |
|  | Second vs third | -58.17 | -131.345 | 15.014 | 0.1281 |
| Social context  df = 2, χ^2^ = 3.573, p = 0.168 | Individual vs unfamiliar | 38.853 | -33.868 | 111.575 | 0.392 |
|  | Individual vs familiar | 54.437 | -18.268 | 127.143 | 0.161 |
|  | Familiar vs unfamiliar | 15.584 | -57.137 | 88.305 | 0.859 |
| Sex × social context | Individual: female vs male | -53.313 | -161.950 | 55.325 | 0.3118 |
|  | Unfamiliar: female vs male | 112.186 | **3.519** | **220.853** | **0.034** |
|  | Familiar: female vs male | -3.188 | -111.825 | 105.450 | 0.952 |
| Social context × sex  df = 2, χ^2^ = 8.185, p = 0.017 | Female: individual vs unfamiliar | -43.90 | -146.720 | 58.928 | 0.5485 |
|  | Female: individual vs familiar | 29.37 | -73.449 | 132.199 | 0.7635 |
|  | Female: familiar vs unfamiliar | 73.27 | -29.553 | 176.094 | 0.1906 |
|  | Male: individual vs unfamiliar | 121.603 | **18.734** | **224.471** | **0.012** |
|  | Male: individual vs familiar | 79.500 | -23.324 | 182.324 | 0.1428 |
|  | Male: familiar vs unfamiliar | -42.103 | -144.971 | 60.766 | 0.576 |

**Table s2**. Output of GLMM with ‘total distance travelled’ as dependent variable (family Gamma, link=log). Effect of ‘part of the day’ (morning or afternoon), ‘round of tests’ (first, second or third), ‘sex’ (female or male), ‘social context’ (individual, unfamiliar, familiar) on total distance travelled. Interaction between social context and sex was excluded as not significant. Fixed effect with significance obtained with ‘Anova’ function in ‘car’ package are presented. Coefficients and 96% confidence intervals are presented; statistically significant comparisons (zero is not included in the interval) are in **bold**. P values obtained with Tukey method adjusted for multiple comparisons.

| **Fixed effect** | **Comparison** | **Estimate** | **2% CI** | **98% CI** | **P value** |
| --- | --- | --- | --- | --- | --- |
| Part of the day  df = 1, χ^2^ = 2.209, p = 0.137 | Morning vs afternoon | -0.210 | 0.500 | -0.080 | 0.137 |
| Sex  df = 1, χ^2^ = 1.536, p = 0.215 | Female vs male | 0.223 | -0.146 | 0.592 | 0.215 |
| Round  df = 2, χ^2^ = 22.452, p <0.0001 | First vs second | -0.445 | **-0.790** | **-0.101** | **0.005** |
|  | First vs third | -0.670 | **-1.022** | **-0.320** | **<.0001** |
|  | Second vs third | -0.225 | -0.572 | 0.121 | 0.255 |
| Social context  df = 2, χ^2^ = 2.671, p = 0.263 | Individual vs unfamiliar | 0.067 | -0.288 | 0.422 | 0.890 |
|  | Individual vs familiar | 0.228 | -0.128 | 0.585 | 0.264 |
|  | Familiar vs unfamiliar | 0.161 | -0.173 | 0.495 | 0.470 |

**Table s3**. Correlation matrix between all dependent variables. Tau values obtained through Kendall Rank correlation. Results in **bold** are significant. False discovery rate correction was applied to value of α.

|  | Areas visited | | | Foraging latency | | |
| --- | --- | --- | --- | --- | --- | --- |
|  | Individual | Unfamiliar | Familiar | Individual | Unfamiliar | Familiar |
| Foraging latency | **-0.233** | -0.075 | -0.086 |  |  |  |
| Ground latency | **-0.452** | **-0.434** | -0.184 | **0.270** | 0.162 | **0.253** |

**Figure s1**. **An example of our test sorting.** All birds performed three tests (individual context, familiar and unfamiliar context). Focal individuals were focal in both their familiar and unfamiliar tests and had two different companions (one they were familiar with and one they were not familiar with). Companion individuals were companions of two different focal individuals, one they were familiar with and one they were not familiar with. Boxes with the same colour (either red or blue) represent sparrows from the same aviary (familiar with each other). Each curved double arrow is a familiar context test, each straight double arrow is an unfamiliar context test, each point is an individual context test. Colours of arrows/points represent the test round: green first round of tests, yellow second round of tests, black third round of tests.

**
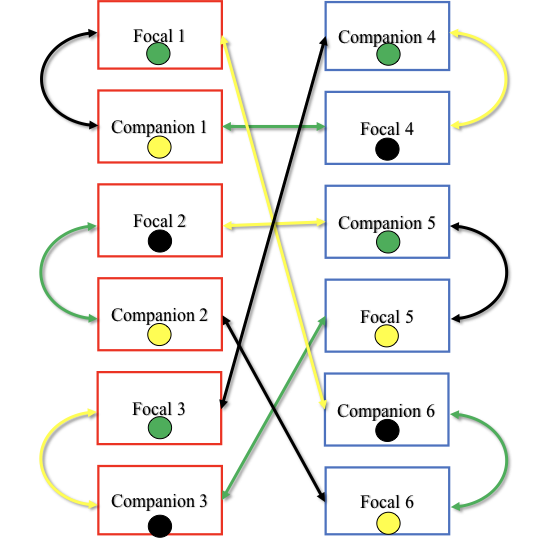
**
